# RNA Methylation in Systemic Lupus Erythematosus

**DOI:** 10.3389/fcell.2021.696559

**Published:** 2021-07-07

**Authors:** Xinyi Lv, Xiaomin Liu, Ming Zhao, Haijing Wu, Wuiguang Zhang, Qianjin Lu, Xiangmei Chen

**Affiliations:** ^1^Hunan Key Laboratory of Medical Epigenomics, Department of Dermatology, The Second Xiangya Hospital of Central South University, Changsha, China; ^2^Department of Nephrology, First Medical Center of Chinese PLA General Hospital, Nephrology Institute of the Chinese People’s Liberation Army, State Key Laboratory of Kidney Diseases, National Clinical Research Center for Kidney Diseases, Beijing Key Laboratory of Kidney Disease Research, Beijing, China

**Keywords:** RNA methylation, SLE, m6A, m5C, immune

## Abstract

Systemic lupus erythematosus (SLE) is an autoimmune disease with complicated clinical manifestations. Although our understanding of the pathogenesis of SLE has greatly improved, the understanding of the pathogenic mechanisms of SLE is still limited by disease heterogeneity, and targeted therapy is still unavailable. Substantial evidence shows that RNA methylation plays a vital role in the mechanisms of the immune response, prompting speculation that it might also be related to the occurrence and development of SLE. RNA methylation has been a hot topic in the field of epigenetics in recent years. In addition to revealing the modification process, relevant studies have tried to explore the relationship between RNA methylation and the occurrence and development of various diseases. At present, some studies have provided evidence of a relationship between RNA methylation and SLE pathogenesis, but in-depth research and analysis are lacking. This review will start by describing the specific mechanism of RNA methylation and its relationship with the immune response to propose an association between RNA methylation and SLE pathogenesis based on existing studies and then discuss the future direction of this field.

## Introduction

Systemic lupus erythematosus (SLE) is a common chronic systemic autoimmune disease that mainly affects young or middle-aged women, with a male-to-female incidence ratio of approximately 1:9 (Stojan and Petri, 2018); men are affected by more severe renal injury (Ramírez Sepúlveda et al., 2019). The incidence rates for SLE range from 0.9 to 7.4 per 100,000 persons/year and differ by sex, region and population (Gergianaki et al., 2017; Stojan and Petri, 2018; Tanaka et al., 2020). For the Asia Pacific region, a meta-analysis showed that the incidence rates of SLE (per 100,000 per year) ranged from 0.9 to 8.4 (Tanaka et al., 2020).

Both environmental factors and genetic susceptibility play vital roles in SLE development. Collectively, evidence has shown that the sustained production of autoantibodies, complement activation, immune complex deposition, neutrophil extracellular traps, lymphocyte signaling and inflammation are all the key mechanisms underlying the loss of tolerance and tissue damage (Tsokos et al., 2016). Patients with SLE can present with diverse organ involvement, such as skin, musculoskeletal, kidney, heart, and lung involvement (Smith and Gordon, 2010; Tsokos et al., 2016). Moreover, patients with SLE have a high overall risk of malignancy (Choi et al., 2017). At present, the early diagnosis of SLE is still based on clinical symptoms and signs, a laboratory examination and biopsy. A recent study compared three classification criteria for evaluating SLE: the European League Against Rheumatism (EULAR)/American College of Rheumatology (ACR)-2019 (Aringer et al., 2019), Systemic Lupus International Collaborating Clinics (SLICC)-2012 (Petri et al., 2012) and ACR-1997 criteria (Hochberg, 1997). An early SLE cohort study found that using these three evaluation criteria might miss or delay the classification and diagnosis of a significant subset of patients with moderate/severe SLE. The general therapeutic approach mainly relies on glucocorticoids and immunosuppressive drugs (ISDs) (Kuhn et al., 2015) with unavoidable side effects, and thus targeted therapy remains an urgent need. Although some progress has been achieved in the early diagnosis and treatment of SLE, the prognosis of patients with SLE is still not optimistic, and the medical cost is high. Therefore, further in-depth exploration of the pathogenesis of SLE is very important for its early diagnosis and the search for new therapeutic targets.

In recent years, an increasing number of scholars have focused their attention on the field of epigenetics, such as DNA methylation, which has led to a better understanding of the pathogenesis of SLE (Xiao and Zuo, 2016). However, with advances in RNA methylation research, scholars have found that RNA methylation is very important in the immune response and is closely related to SLE. RNA, an important intermediate product of gene expression, is also regulated by chemical modification after transcription and during translation through a process collectively referred to as epitranscriptomics (Saraceno et al., 2016). Similar to chemical modifications of DNA and histones, chemical modifications of RNA also regulate gene expression through mechanisms such as changing the structural properties of RNA or changing the affinity of mRNA for ribosomes. The discovery, continuous exploration, and in-depth study of chemical modifications in RNA have led epigenetic research to a new level and provided a new direction for further exploring the pathogenesis of diseases and developing new therapeutic strategies.

Therefore, this article will start by describing RNA methylation, summarize the application of RNA methylation in immunology, and speculate on the relationship between RNA methylation and the pathogenesis of SLE to provide more ideas for future research.

## The Specific Mechanism of RNA Methylation

Chemical modifications of cellular RNAs are natural and abundant. The dynamic nature and increasing number of RNA modifications provide new possibilities for rapidly altering gene expression to adapt to specific environments. Due to the development of genomic approaches, various modifications on RNA have been identified and investigated. As of 2017, more than 150 distinct chemical modifications on RNA have been detected (Boccaletto et al., 2018).

N^6^-methyladenosine (m6A) is the most widespread and investigated modification of mammalian mRNA and has a broad range of critical functions in development (Kasowitz et al., 2018), cancer (Ma S. et al., 2019), and viral infection (Williams et al., 2019). Pseudouridine (Ψ) was the first structurally modified nucleoside identified in the 1950s, and is known as the fifth nucleoside (Cohn and Volkin, 1951). Ψ generates an extra hydrogen bond donor at the newly formed N1 position, which increases the structural stability of the RNA and translation efficiency and accuracy (McKenney et al., 2017). The presence of N1mΨ (N1-methyl-pseudouridine (N1mΨ) in mRNA favors ribosome recycling on the same mRNA or *de novo* ribosome recruitment (Svitkin et al., 2017). 5-Methylcytidine (m5C) has long been studied in DNA. In RNA, m5C levels are 3–10-fold rarer than m6A levels (Legrand et al., 2017). Although the biological function of m5C in eukaryotic mRNA is just beginning to become clear, it is postulated to have a powerful function in regulating cellular processes (Trixl and Lusser, 2019).

Three types of molecules are involved in RNA methylation: writers, erasers and readers. Writers, namely, methyltransferases, transfer methyl groups to RNA in the form of protein complexes, individual proteins of which might have specific functions or integrate different signals. Erasers, namely, demethylases, erase the RNA methylation modification to convert m6A into RNA. Readers recognize the RNA methylation modification information to guide and participate in the translation and degradation of the modified downstream RNA sequence. In general, RNA methylation is first performed by writer complexes at different RNA sites and then can be demethylated by erasers, which makes RNA methylation a reversible process. The modified base sites are recognized by specific readers to mediate specific biological functions. Readers located in the nucleus might affect mRNA splicing or other nuclear processes, while those located in the cytoplasm might affect the stability, translation, or location of mRNAs. These three types of molecules are indispensable to achieving the regulatory functions of RNA methylation. We list the most well-studied writers, erasers and readers for m6A in Table 1 and m5C in Table 2.

**TABLE 1:** Writers, erasers, and readers for m6A.

**Effect**	**Protein name**	**Cellular location**	**Effect on RNA methylation and its mechanisms**	**Evidence related to SLE**
Writers (methyltransferase)	METTL3/MTA70	Nucleus and cytosol	The central methyltransferase that installs m6A residues on mRNAs and lncRNAs in eukaryotes Lin et al., 2019	The levels of the METTL3 mRNA are significantly decreased in the peripheral blood of patients with SLE compared with healthy controls Luo et al., 2020a.
	METTL5		Mediates the m6A modification of human 18S rRNA with the activation of TRMT112 van Tran et al., 2019; Ignatova et al., 2020	–
	METTL14	Nucleus and cytosol	Forms a stable heterodimer with METTL3 and enhances the methylation activity of METTL3	The expression of the METTL14 mRNA is decreasing in patients with SLE compared with healthy controls, which was associated with white blood cell count and monocyte count Luo et al., 2020b
	WTAP	Nucleus	Changes the alternatively spliced mRNA model Ping et al., 2014; shows no methyltransferase activity, but potentially enhances methyltransferase activity of the METTL3-METTL14 heterodimer Liu et al., 2014	The levels of the WTAP mRNA are significantly decreased in the peripheral blood of patients with SLE compared with healthy controls Luo et al., 2020a.
	TRMT112		Forms a stable heterodimer with METTL5 and enhances the methylation activity of METTL5 van Tran et al., 2019; Ignatova et al., 2020	–
	VIRMA/KIAA1429		Assembles core components through its N-terminus Yue et al., 2018	–
	RBM15/15b		Mediates the m6A modification of lncRNA-XIST Patil et al., 2016.	–
	ZCCHC4		Mediates the m6A modification of human 28S rRNA Ma H. et al., 2019; Ren et al., 2019; Pinto et al., 2020	–
	ZC3H13	Nucleus	Improves the catalytic function of WTAP and MTC, which are retained in nuclear speckles, by interacting *via* its low-complexity (LC) domains Knuckles et al., 2018; Wen et al., 2018	–
	CBLL1	Nucleus	Assists in the activation of WTAP by forming stable interactions Figueroa et al., 2009	–
Erasers (demethylase)	FTO	Mainly in the nucleus	Removes m6A from mRNA and m1A from tRNA through its C-terminus Jia et al., 2011	The levels of the FTO mRNA are significantly decreased in the peripheral blood of patients with SLE compared with healthy controls Luo et al., 2020a
	ALKBH5	Mainly in the nucleus	Removes m6A from mRNA Zheng et al., 2013.	The levels of ALKBH5 mRNA in the peripheral blood of patients with SLE are related to anti-dsDNA antibodies, antinucleosome antibodies, rash, and ulceration. Based on this evidence, the ALKBH5 mRNA level might be involved in the pathogenesis of SLE Luo et al., 2020a, b.
Readers (methylation recognition protein)	YTHDC1	Nucleus	Stimulates splicing and mRNA export (direct binding to m6A) Roundtree and He, 2016; Roundtree et al., 2017; reduces the rRNA synthesis Chen et al., 2021	–
	YTHDC2	Nucleus and cytosol	Stimulates mRNA decay and translation (direct binding to m6A) Hsu et al., 2017; Wojtas et al., 2017	–
	YTHDF1	Cytosol	Stimulates translation (direct binding to m6A) Wang et al., 2015	–
	YTHDF2	Cytosol	Stimulates RNA decay and translation (direct binding to m6A) Wang et al., 2014	The levels of YTHDF2 mRNA are decreased in peripheral blood from patients with SLE Luo et al., 2020a, b, which might be risk factors for SLE Luo et al., 2020b.
	YTHDF3	Cytosol	Stimulates RNA decay and translation (direct binding to m6A) Li A. et al., 2017; Shi et al., 2017.	–
	HNRNPA2B1	Nucleus	Mediates m6A-dependent miRNA processing and may affect splicing (binding regulated by m6A-induced structural changes) Alarcón et al., 2015	–
	HNRNPC	Nucleus	Affects mRNA splicing (binding regulated by m6A-induced structural changes) Liu et al., 2015	–
	IGF2BP1-3	Nucleus and cytosol	Increases mRNA stability (binding regulated by m6A-induced structural changes) Huang et al., 2018	–
	FMRP	Nucleus and cytosol	Directly or indirectly maintains the stability of m6A-containing mRNAs by directly binding to YTHDF2 (binding to bona fide m6A-binding proteins) Edupuganti et al., 2017; Huang et al., 2018	–

*WTAP, Wilms’ tumor 1-associated protein; TRMT112, tRNA methyltransferase 11-2 (TRMT112); VIRMA/KIAA1429, vir-like m6A methyltransferase-associated protein; RBM15/15b, RNA-binding motif protein 15/15b; ZCCHC4, zinc finger CCCH-type containing 4; ZC3H13, zinc finger CCCH-type containing 13; CBLL1, Cbl proto-oncogene like 1; FTO, obesity-associated protein; HNRNPA2B1, heterogeneous nuclear ribonucleoproteins A2B1; HNRNPC, heterogeneous nuclear ribonucleoproteins C; IGF2BP1-3, insulin-like growth factor 2 mRNA-binding protein1-3; FMRP, fragile X mental retardation protein.*

**TABLE 2:** Writers, erasers, and readers for m5C.

**Effect**	**Protein name**	**Cellular location**	**Effect on RNA methylation and its mechanisms**	**Evidence related to SLE**
Writers (methyltransferase)	DNMT2/TRDMT1	Nucleus and cytosol	Mainly mediates the m5C modification of DNA and tRNA Raddatz et al., 2013	–
	NSUN2	1. G1 phase: nucleolus 2. S phase: between nucleolus and nucleoplasm 3. G2 phase: cytoplasm, M phase: centrioles	Modifies some non-coding small RNAs and tRNA Khoddami and Cairns, 2013	The expression of NSUN2 was decreasing in CD4^+^ T cells from patients with SLE compared with healthy controls Guo et al., 2020.
	NSUN5		Mediates the m5C modification of human 28S rRNA Janin et al., 2019	–
	TRM4B		Involved in tRNA methylation and recognition Cui X. et al., 2017; David et al., 2017	–
Erasers (demethylase)	TET2	Nucleus	Involved in 5-methylcytidine oxidation Xue C. et al., 2020	Silencing of the TET2 gene obviously diminishes follicular helper T cell polarization *in vitro*, which plays a critical role in SLE Wu et al., 2016.
Readers (methylation recognition protein)	ALYREF	Nucleus	Involved in mRNA nuclear-cytoplasmic shuttling, viral RNA export and replication Xue C. et al., 2020	
	Cytoplasmic YBX1	Cytoplasm	Involved in mRNA stabilization, embryogenesis and tumorigenesis Xue C. et al., 2020	
	TRM4B		Involved in tRNA methylation and recognition Cui X. et al., 2017; David et al., 2017	

*DNMT2/TRDMT1, DNA methyltransferase E2/tRNA aspartic acid methyltransferase 1; NSUN2, NOL1/NOP2/SUN domain methyltransferase family2; NSUN5, NOL1/NOP2/SUN, domain methyltransferase family5; TRM4B, tRNA-specific methyltransferase 4B; TET2, ten-eleven translocation 2; ALYREF, Aly/REF output factor; YBX1, Y-box binding protein 1.*

Studies have identified important roles for m6A and m5C in the development and regulation of many organs and systems, especially in the immune system. Therefore, we should focus on the specific mechanism of RNA methylation at m6A and m5C and the potential mechanism in the pathogenesis of SLE.

### m6A

The methylation of the sixth position of the RNA adenine ring and occurs in the sequence context Pu[G > A]m6AC[U > A > C](Pu = purine) (Schibler et al., 1977). It was first discovered in 1974 (Desrosiers et al., 1974) and plays a conservative role in the evolution of meiosis and cell differentiation in yeast, plants and mammals (Yue et al., 2015). Although m6A may exist in the primary transcript, in mammals and yeast, m6A is mainly located in genes, namely, the mRNA protein coding region (CDS) near the termination codon and 3′ untranslated region (3′ UTR) (Dominissini et al., 2012; Ke et al., 2015). Coding RNAs and non-coding RNAs, including tRNAs, rRNAs, small nuclear RNAs, microRNA (miRNA) precursors and long non-coding RNAs (lncRNAs), are modified with m6A in a variety of tissues.

#### m6A Writers

The m6A methyltransferase complex transfers a methyl group from the donor substrate S-adenosyl methionine (SAM) to adenine nucleotides in the recipient RNA subunit (Bokar et al., 1997). The complex consists of METTL3 (also known as MTA70) (Lin et al., 2019), METTL5 (van Tran et al., 2019; Ignatova et al., 2020), METTL14 (Yue et al., 2015; Liu et al., 2016), Wilms’ tumor 1-associated protein (WTAP) (Ping et al., 2014), tRNA methyltransferase 11-2 (TRMT112) (van Tran et al., 2019; Ignatova et al., 2020), vir-like m6A methyltransferase-associated protein (VIRMA, originally known as KIAA1429) (Yue et al., 2018), RNA-binding motif protein 15/15b (RBM15/15b) (Patil et al., 2016), zinc finger CCCH-type containing 4 (ZCCHC4) (Ma H. et al., 2019; Ren et al., 2019; Pinto et al., 2020), zinc finger CCCH-type containing 13 (ZC3H13) (Knuckles et al., 2018; Wen et al., 2018), and Cbl proto-oncogene like 1 (CBLL1) (Figueroa et al., 2009), among which the most common molecular components are METTL3 and METTL14.

METTL3 is considered the central methyltransferase because of its ability to bind SAM, and it is highly conserved in eukaryotes (Lin et al., 2019). The discovery of METTL3 initiated research on the relationship between m6A and cellular physiology. Differences in the METTL3 expression levels reflect changes in the total m6A level. METTL3 can be modified by SUMO1 at lysine residues K^177^, K^211^, K^212^, and K^215^, significantly repressing the methyltransferase activity of METTL3 (Du et al., 2018). SUMOylation is a reversible posttranslational modification process that attaches small ubiquitin-like modifiers to protein substrates (Hay, 2005). Moreover, sentrin/SUMO-SPECIFIC PROTEASE 7 is significantly upregulated in patients with SLE (Cui Y. et al., 2017), and levels of the METTL3 mRNA are significantly decreased in the peripheral blood of patients with SLE compared with healthy controls (Luo et al., 2020a). Thus, we speculate that SUMOylation might play roles in reducing the expression of METTL3 in patients with SLE.

METTL14 is highly homologous to METTL3. It forms a stable heterodimer with METTL3, enhancing the methylation activity of METTL3. Together, the heterodimer of METTL3-METTL14 forms the catalytic core of the m6A methyltransferase complex (Yue et al., 2015; Liu et al., 2016). Sanna Bystrom found that the expression profile of the METTL14 protein was altered in patients with multiple sclerosis (Byström et al., 2014). Qing Luo et al. observed decreased expression of the METTL14 mRNA in patients with SLE compared with healthy controls (*p* < 0.001), which was associated with white blood cell count and monocyte count (Luo et al., 2020b). Evidence also shows that the production of type 1 interferon (IFN I), the most important cytokine involved in SLE pathogenesis, its production triggered by dsDNA or human cytomegalovirus is controlled by cellular METTL14 and ALKBH5 demethylases (Rubio et al., 2018). METTL14 depletion increases both the production and stability of the nascent IFN β1 mRNA in response to dsDNA (Rubio et al., 2018). This phenomenon represents a potential mechanism by which METTL14 participates in the development of autoimmune diseases, especially SLE.

WTAP is also a core component of the m6A methyltransferase complex that interacts with METTL3 and METTL14 (Ping et al., 2014). The intracellular m6A abundance was markedly decreased when WTAP was knocked out compared with METTL3 or METTL14, which might result from changes in the alternative splicing of the mRNAs to which WTAP binds (Ping et al., 2014). Liu et al. found that the m6A level in polyadenylated RNA was decreased by ∼30, ∼40, and ∼50% in HeLa cells with knockdown of cellular METTL3, METTL14, and WTAP, respectively (Liu et al., 2014). Moreover, WTAP itself showed no methyltransferase activity but dramatically enhanced the methyltransferase activity when interacting with the METTL3-METTL14 heterodimer (Liu et al., 2014). WTAP is upregulated in many tumors, where it functions as an oncogene by interacting with different proteins involved in RNA processing (Sorci et al., 2018).

#### m6A Erasers

The m6A demethylase is responsible for the selective removal of SAM from the adenine nucleotides of RNA to regulate gene expression and cell fate. Fat mass and obesity-associated protein (FTO) was the first RNA demethylase discovered, and its C-terminal structure demethylates mRNA, mainly in the nucleus (Jia et al., 2011; Bartosovic et al., 2017). Several studies have shown that FTO might not have a physiological function toward m6A because the FTO knockout transcriptome does not contain an increased number of m6A sites (Hess et al., 2013; Mauer et al., 2017; Garcia-Campos et al., 2019). Instead, FTO appears to function in specific tissues or under specific conditions. For example, FTO regulates the expression of oncogenes, namely, an ∼20% increase, by reducing the m6A modification in mRNA to enhance leukemic oncogene-mediated cell transformation and leukemogenesis (Li Z. et al., 2017). As shown in the study by Qing Luo, FTO expression positively correlates with SLE in patients. The mRNA levels of FTO in the peripheral blood of patients with SLE are significantly decreased compared with those of healthy controls (Luo et al., 2020a).

ALKBH5, the second RNA eraser identified, was shown to affect mouse spermatogenesis (Zheng et al., 2013). ALKBH5 is primarily colocalized with nuclear speckles and affects mRNA export and RNA metabolism in a demethylation-dependent manner (Zheng et al., 2013). Rubio et al. (2018) found that IFN I production triggered by dsDNA or human cytomegalovirus is affected by ALKBH5. ALKBH5 depletion reduces nascent IFN β1 mRNA production without detectably influencing IFN β1 mRNA decay (Rubio et al., 2018). Moreover, the levels of ALKBH5 mRNA in the peripheral blood of patients with SLE are related to anti-dsDNA antibodies, antinucleosome antibodies, rash and ulceration (Luo et al., 2020a). Based on this evidence, the ALKBH5 mRNA level might be involved in the pathogenesis of SLE (Luo et al., 2020a).

#### m6A Readers

The downstream function of the presence of m6A on mRNA molecules is closely related to the recognition and binding of m6A-methylated recognition proteins, termed m6A readers. Various m6A readers have been identified, but their mechanism is similar: m6A readers recognize and bind the RNA decorated by m6A. The regulatory function of m6A is achieved by enhancing or weakening the recruitment of different RNA-binding proteins (RBPs) to target mRNAs or directly inducing secondary structural changes in target mRNAs to influence the interaction between RNAs and RBPs (Adhikari et al., 2016; Wu B. et al., 2017).

The most important m6A recognition protein in eukaryotes is the YTH domain-containing family proteins, which comprise the conserved C-terminal RNA recognition and binding domain YTH and the N-terminal variable region. This protein family is also considered the most primitive m6A reader, falling into three classes: YTHDC1, YTHDC2, and the YTHDF family (Zhang et al., 2010). Among them, YTHDF1, YTHDF2, and YTHDF3, which exist in humans, have been studied more extensively and have different cellular localizations but similar functions (Liao et al., 2018; Patil et al., 2018). Other recognized m6A recognition proteins include heterogeneous nuclear ribonucleoprotein A2B1 (HNRNPA2B1) (Alarcón et al., 2015), heterogeneous nuclear ribonucleoproteins C (HNRNPC) (Liu et al., 2015), fragile X mental retardation protein (FMRP) (Edupuganti et al., 2017) and insulin-like growth factor 2 mRNA-binding protein1-3 (IGF2BP1-3) (Huang et al., 2018).

#### Effects of m6A Methylation

The m6A modification on RNA regulates the transcription of genes to achieve functional regulation at the cellular or tissue level, which is mainly achieved by the functions of various m6A methyltransferases, m6A demethylases and m6A recognition proteins. m6A is mainly modified on mRNA, and thus the main effects of the m6A modification include pre-mRNA shearing, the stability of mRNA, nuclear transfer of mRNA, and translation of mRNA, thus achieving the regulation of mRNA function (Meyer et al., 2015; Gan et al., 2019; Zhang et al., 2019a). For example, protein translation typically begins with the recruitment of the 43S ribosomal complex to the 5′ cap of mRNAs by a cap-binding complex. Evidence has shown that mRNAs containing m6A in their 5′ UTR are translated in a cap-independent manner (Meyer et al., 2015).

In addition, m6A also modifies non-coding RNAs, such as rRNA, miRNAs, and lncRNAs. m6A methylation of rRNA is dispensable in cell growth and ribosome biogenesis, but plays important roles in increasing translation efficiency and cell proliferation and differentiation (van Tran et al., 2019). Increasing translation efficiency is achieved by the regulation of the kinetics of translation rather than rRNA processing (van Tran et al., 2019; Pinto et al., 2020). Some researchers an increasing polysome/monosome ratio *via* m6A methylation of rRNA (Ma H. et al., 2019), but this phenomenon has not been widely recognized in other studies (van Tran et al., 2019; Pinto et al., 2020). The positive effects on cell proliferation and differentiation have been shown by knocking down the m6A writers of rRNA (Ma H. et al., 2019; Ignatova et al., 2020; Xing et al., 2020). High m6A methylation levels in rRNA have also been found in cancer (Ma H. et al., 2019). Meanwhile, METTL3 may play a positive role in prerRNA processing by influencing relevant protein expression or modifying snoRNA (Sergeeva et al., 2020). The regulation of m6A methylation is also be embodied in premiRNA processing and lncRNA processing. Meanwhile, by sequence pairing with mRNAs containing miRNA target sites, miRNAs regulate the binding of METTL3 to target RNAs, leading to an increase in the m6A modification (Fazi and Fatica, 2019).

### m5C

m5C is the best-understood epigenetic modification of DNA (Jones, 2012) and is also present in RNAs that are more diverse and complex (Gilbert et al., 2016) The m5C methyltransferase catalyzes the methylation of the 5th position of the cytosine pyrimidine ring at a specific site (Schosserer et al., 2016). The function of m5C has been unclear for many years. However, with the continuous development of high-throughput sequencing technology, the specific locations and related functions of m5C have gradually become clear. Researchers have found that m5C is distributed across coding RNA sequences and all types of non-coding RNAs, such as tRNA and rRNA (Squires et al., 2012; Gilbert et al., 2016). Moreover, the m5C modification of RNA is an important regulator of many aspects of gene expression, including RNA processing and degradation, ribosomal assembly, translation, and RNA stability (Tuorto et al., 2012; Blanco et al., 2016; Nakano et al., 2016).

#### m5C Writers

Among higher eukaryotes, the most frequently studied m5C methyltransferases are DNA methyltransferase E2/tRNA aspartic acid methyltransferase 1 (DNMT2/TRDMT1) (Tang et al., 2003) and the NOL1/NOP2/SUN domain methyltransferase family (NSUN) (Bohnsack et al., 2019). Mechanistically, both DNMT2 and NSUN form covalent intermediates through the interaction of a cysteine and cytosine of the target RNA to promote nucleophilic attack at C5 of the pyrimidine ring by SAM to form the m5C modification. However, the difference is that a single cysteine in DNMT2 molecules forms a covalent intermediate, with cytosine Myc-induced SUN domain-containing protein (Misu/NSUN2) molecules formed by two cysteine and cytosine in the covalent intermediate (King and Redman, 2002).

DNMT2 is a widely conserved member of the eukaryotic cytokine-5-DNA methyltransferase protein family, which is widely distributed in the nucleus and cytosol (Tang et al., 2003). Although DNMT2 mainly mediates m5C methylation in DNA, many studies have recently shown that DNMT2 could mediate the m5C modification of tRNA (Raddatz et al., 2013; Genenncher et al., 2018). According to recent studies, DNMT2 mainly mediates the methylation of tRNA at C38 in eukaryotic cells (Schaefer et al., 2010), and DNMT2-mediated RNA methylation has been detected in some eukaryotic organisms, such as zebrafish (Rai et al., 2007) and *Drosophila* (Schaefer et al., 2010). DNMT2-mediated RNA methylation exerts an important effect on organ differentiation and environmental tolerance (Schaefer et al., 2010). Evidence has shown that DNMT2 is required for an efficient innate immune response in *Drosophila*, which is possibly mediated by RNA methylation (Durdevic et al., 2013).

NSUN2 is a member of the protein family containing the NOL1/NOP2/SUN domains, which are mainly located in the nucleus. NSUN2 modifies some non-coding small RNAs and mRNAs in addition to tRNA (Khoddami and Cairns, 2013; Li Q. et al., 2017; Yang et al., 2017). A recent study showed substantially reduced NSUN2 expression levels in CD4^+^ T cells from patients with SLE compared with healthy controls, which might be due to RNA methylation (Guo et al., 2020).

tRNA-specific methyltransferase 4B (TRM4B) is also an important m5C methyltransferase, and it has been proven to be relevant to tRNA methylation and root growth in *Arabidopsis* (Cui X. et al., 2017; David et al., 2017), but this result still needs to be confirmed in mammalian cells.

#### m5C Erasers

The modification of m5C is reversible. Recent studies identified the m5C demethylase ten-eleven translocation 2 (TET2) (Wu and Zhang, 2011; Xue M. et al., 2020). Silencing of the TET2 gene obviously diminishes follicular helper T cell polarization *in vitro*, which plays a critical role in SLE. Because TET2 functions in both DNA demethylation and RNA demethylation, the specific mechanism of m5C in SLE deserves further study (Wu et al., 2016).

#### m5C Readers

To date, three m5C-binding proteins have been identified as m5C readers: Aly/REF output factor (ALYREF, an mRNA transport adaptor) (Yang et al., 2017), cytoplasmic Y-box binding protein 1 (YBX1), and TRM4B (Cui X. et al., 2017; Chen X. et al., 2019). The ALYREF-dependent pathway potentially represents one of the main mechanisms for the selective export of m5C-modified mRNAs in mammals (Yang et al., 2017).

#### Effects of m5C Methylation

Similar to m6A methylation, the effect of m5C is mainly achieved by influencing the process of protein translation. Studies have shown that m5C induces ribonuclease activity to promote tRNA degradation and affect protein translation; moreover, the modification of m5C on rRNA also affects protein translation (Burgess et al., 2015), and some experiments have suggested that m5C affects the stability of mRNA (Hussain et al., 2013). Although a variety of molecules are modified with m5C, in eukaryotes, the m5C modification is mainly detected on tRNA (Squires et al., 2012). With the exception of tRNA^Leu CAA^, the m5C modification of tRNA mainly occurred outside anticodon rings (Chan et al., 2012). Therefore, the main effect of the CAA m5C modification is to regulate the translation efficiency by affecting CAA oscillation (Chan et al., 2012), while the m5C modification outside the anti-codon ring mainly affects the structure and stability of tRNA for the purpose of regulation (Vare et al., 2017; Janin et al., 2019). By regulating protein translation, m5C also plays a role in many normal physiological processes and abnormal diseases.

## Potential Links Between RNA Methylation and Sle

### RNA Methylation in T Cells

**FIGURE 1:**
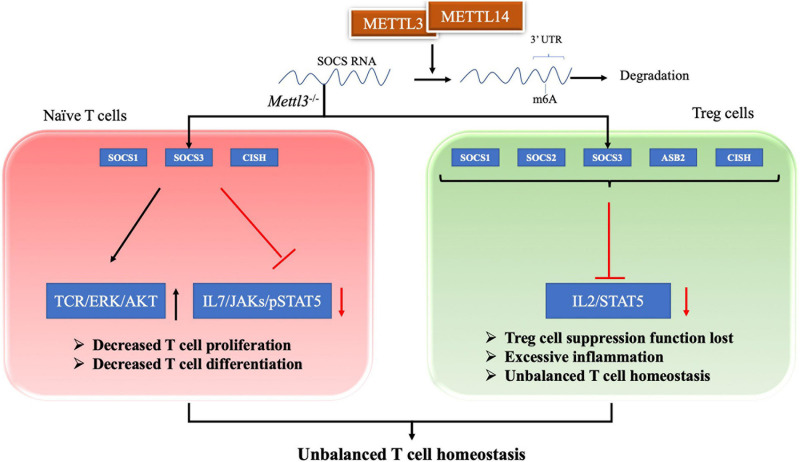
RNA methylation in T cell homeostasis. For naive T cell, SOCS family genes with m6A modification exhibit slower mRNA decay and increased protein expression levels. The increased SOCS family activity inhibits the IL-7/STAT5 signaling pathway and activates the TCR/ERK/AKT pathway, which lead to decreased T cell proliferation and differentiation. For Treg cell, reduction in m6A enhances the mRNA stability of SOCS genes and the proteins levels. The increased SOCS family activity blocks signal transduction of the IL2-STAT5 pathway, which lead to Treg cell suppression function lost. These two evidences shows that m6A modification plays an important role in maintaining T cell homeostasis.

T cells are regarded as a central component of the pathogenesis of SLE (Tsokos et al., 2016). T cell homeostasis is the key process in maintaining the T cell pool size, and its imbalanced state is essential in the pathogenesis of SLE (Oster et al., 2019; Katsuyama et al., 2020). Evidence has shown that the m6A modification plays an important role in maintaining T cell homeostasis. Li HB and colleagues found that the m6A modification controls the differentiation of naïve T cells (Li H.-B. et al., 2017). Conditioned knockout of the *Mettl3* gene in mouse CD4^+^ T cells reduces the m6A methylation level in naïve T cells, which leads to an increase in Th2 cells and a decrease in Th1 and Th17 cells, but has no effect on cell apoptosis or TCR-mediated proliferation (Li H.-B. et al., 2017). Researchers have inferred that naïve T cells lacking METTL3 or METTL14 does not undergo homeostatic amplification and remain naïve, mainly because SOCS family genes (*Socs1*, *Socs3*, and *Cis*) with less m6A modification exhibit slower mRNA decay and increased protein expression levels (Li H.-B. et al., 2017). Therefore, the increased SOCS family activity inhibits the IL-7/STAT5 signaling pathway and activates the TCR/ERK/AKT pathway, which inhibits T cell proliferation and differentiation (Palmer and Restifo, 2009; Li H.-B. et al., 2017). Afterward, RNA methylation was also shown to regulate T cell homeostasis through a repressive loss of function of regulatory T cells (Tregs) (Tong et al., 2018). The authors generated *Mettl3*^f/f^; Foxp3Cre mice to determine the role of m6A methylation in T cell homeostasis *in vivo*. Inflammatory Th1 and Th7 responses were significantly increased in spleen and peripheral lymph nodes compared with wild-type mice at 60 days after birth (Tong et al., 2018). In *Mettl3*^–/–^ Tregs, the reduction in m6A levels increases the mRNA stability of *SOCS* genes, including *Cish, Socs1, Socs2, Socs3*, and *Asb2*, and increased levels of SOCS proteins block signal transduction from the IL2-STAT5 pathway, which is essential for Treg function and stability (Shi et al., 2018; Tong et al., 2018). Thus, a loss of Treg function eventually leads to excessive inflammation, inhibiting the function of Tregs and playing an important role in regulating T cell homeostasis (Li and Rudensky, 2016; Tong et al., 2018). In addition to METTL3, METTL14 deficiency in T cells also induces unbalanced T cell homeostasis. A METTL14 deficiency in T cells induces spontaneous colitis in mice, manifesting as increased inflammatory cell infiltration and cytokine production from Th1 and Th17 cells (Lu T. X. et al., 2020). The Mettl14 deficiency also caused impaired induction of the differentiation of naïve T cells into induced Tregs (Lu T. X. et al., 2020). Therefore, the m6A modification exerts a positive regulatory effect on T cell differentiation and development and is one of the important regulatory mechanisms of adaptive immunity, as shown in Figure 1.

Evidence related to the role of m5C in T cells is limited. The m5C modification stabilizes mRNA expression, leading to an imbalance in Th17/Treg differentiation, which may reveal the potential mechanism of m5C in SLE pathology (Guo et al., 2020). The specific mechanism is worth further exploration.

### RNA Methylation in Dendritic Cells

Dendritic cells (DCs) play important roles in the innate and adaptive immune responses, as well as in the progression of SLE (McHugh, 2019; Xiao et al., 2020). A study revealed that DCs exposed to m6A- or m5C-modified nucleosides expressed significantly fewer cytokines and activation markers than those treated with unmodified RNA (Karikó et al., 2005). Mammalian total RNA, but not bacterial RNA, is abundant in modified nucleosides (Karikó et al., 2005). The innate immune system selectively responds to bacteria or necrotic tissue by detecting RNA lacking modification (Karikó et al., 2005). However, METTL3-mediated m6A methylation in DCs is required for the activation and function of DCs. METTL3 induces the phenotypic and functional maturation of DCs, and the expression of CD40, CD80, and IL-12 and the ability to stimulate T cells both *in vitro* and *in vivo* are promoted in *Mettl*3KO DCs (Wang H. et al., 2019). As shown in the study by Han D., knockdown of YTHDF1, an m6A-binding protein, in classic DCs enhanced the antitumor immune response *in vivo* by enhancing the cross-presentation of tumor antigens and the cross-priming of CD8^+^ T cells (Han et al., 2019). Thus, RNA methylation also regulates the function of DCs in the immune response.

### RNA Methylation in Inflammation

RNA methylation is also an essential mechanism in the process of inflammation, and it is a key component of the pathogenesis of SLE (Frangou et al., 2019). The dynamic regulation of these inflammatory factors contributes to the susceptibility to SLE but is more strongly implicated in the loss of tolerance and end organ effects (Hu et al., 2015). Patients with SLE usually present increased serum levels of inflammatory factors, such as IL-6, TNF-α, and IL-1β, which are related to various immune processes in SLE pathogenesis (Wu Y. et al., 2017; Paradowska-Gorycka et al., 2019; Uzrail et al., 2019). For example, the elevated levels of IL-6 and TNF-α expression in PBMCs from patients with SLE are closely associated with the Th1/Th2/Th17 inflammatory response, which is positively correlated with SLE disease activity (De la Cruz-Mosso et al., 2018). Increased levels of IL-6, IL-10, and TNF-α are consistent with B cell proliferation and activation in patients with SLE (Fleischer et al., 2015). Thus, RNA methylation participates in SLE pathogenesis by regulating inflammatory factor expression, but further evidence is needed. To date, *in vitro* and *in vivo* studies have shown that RNA methylation regulates the occurrence of inflammation through several signaling pathways.

First, the level of RNA methylation regulates the expression of inflammatory factors through the MyD88 pathway. METTL3 knockdown decreases the lipopolysaccharide-induced expression of inflammatory cytokines, including IL-6, IL-8, GRO, Gro-α and RANTES (Feng et al., 2018; Zhang et al., 2019b), by facilitating the expression of MyD88S, a splice variant of myeloid differential protein-88 (MyD88) (Feng et al., 2018). Moreover, RNA methylation regulates inflammation by influencing the NF-κB and MAPK signaling pathways through effects on the phosphorylation of related molecules. METTL3 knockdown decreases the phosphorylation of IKKα/β, p65 and IκBα in the NF-κB signaling pathway and p38, ERK, and JNK in the MAPK signaling pathway in LPS-induced inflammation (Feng et al., 2018; Zhang et al., 2019b). Analogously, YTHDF2 knockdown increases the phosphorylation of p65, p38 and ERK1/2 in the NF-κB and MAPK signaling pathways (Yu et al., 2019). In addition, RNA methylation of the forkhead box O (FOXO) mRNA is also involved in the inflammation process. METTL14 and METTL3 separately increase m6A methylation of the FOXO1 and FOXO3 mRNAs and increase their stability *via* the interaction of YTHDF1 (Jian et al., 2020; Lin et al., 2020). YTHDF3 promotes FOXO3 translation by binding to the translation initiation region of the FOXO3 mRNA (Zhang et al., 2019c). As an important transcription factor, FOXO1 promotes the expression of VCAM-1 and ICAM-1 by directly binding to their promoter regions, which leads to endothelial inflammation and atherosclerosis development (Jian et al., 2020). The FOXO3 levels in B cells from patients with SLE are inversely correlated with disease activity and reduced in patients with elevated anti-dsDNA Ab levels (Ottens et al., 2018).

The regulatory effect on these inflammasome pathways in different autoimmune diseases has been reported. *In vitro* and *in vivo* experiments using samples from patients with rheumatoid arthritis (RA) revealed significantly elevated METTL3 levels in patients with RA that played an important role in LPS-induced inflammation in macrophages *via* the NF-κB signaling pathway (Wang J. et al., 2019). In the development of osteoarthritis (OA), METTL3 expression increases and subsequently regulates inflammation *via* the NF-κB signaling pathway and the degradation of extracellular matrix (ECM) (Liu et al., 2019). These findings not only provide new insights into the pathogenesis of SLE but also facilitate the identification of therapeutic targets and provide new directions for future research. For example, oleuropein (OL) regulates the activation of the JAK/STAT, MAPK, NF-κB and NLRP3 inflammasome pathways and exert its therapeutic effect on patients with SLE (Castejon et al., 2019).

### Role of RNA Methylation in IFN I Production

IFN I is a vital component of the antiviral innate immune response and is also the most important cytokine involved in SLE pathogenesis. We outline the studies that revealed the role of RNA methylation in IFN I production to determine the potential correlation between RNA methylation and SLE.

**FIGURE 2:**
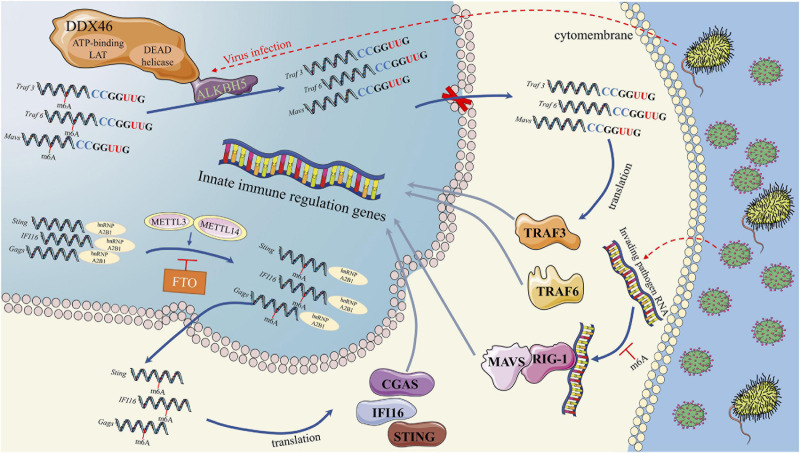
RNA methylation in Interferon production in process of virus recognition. RNA methylation in Interferon production were mainly through three pathways. First, DDX46 and ALKBH5 complex could demethylate m6A-modified antiviral transcripts, which lead to antiviral transcript retention in the nucleus and decreases the IFN I protein level. Second, HNRNPA2B1 could activates the TBK1-IRF3 pathway by binding to CGAS, IFI16, and STING to promote IFN I production. METTL3 and FTO could regulate the function of HNRNPA2B1 by m6A methylation. Third, m6A modified transcripts could interrupts the RIG-I like innate immune activation, which could mediate the activation of transcription factors and interferon-stimulated genes.

First, the m6A modification plays an important role in the regulation of IFN I production during virus recognition (Figure 2). The dead-box (DDX) helicase family plays an important role in identifying viral nucleic acids and regulating downstream pathways (Parvatiyar et al., 2012). DDX3 and DDX46 recruit and interact with ALKBH5 *via* the DEAD helicase domain (Shah et al., 2017; Zheng et al., 2017). The complex of DDX46 and ALKBH5 demethylates m6A-modified antiviral transcripts, which increases antiviral transcript (Mavs, Traf3, and Traf6) retention in the nucleus and decreases the expression of these proteins and IFN I (Zheng et al., 2017). In addition to DDX46, the function of HNRNPA2B1, a DNA virus sensor, in activating the TANK-binding kinase 1–interferon regulatory factor 3 (TBK1–IRF3) pathway and subsequent IFN-α/β production is also regulated by the m6A modification (Wang L. et al., 2019). In the cytoplasm, HNRNPA2B1 activates the TBK1-IRF3 pathway by binding to CGAS, IFI16, and STING to promote IFN I production (Wang L. et al., 2019). In this process, an RNA writer (METTL3) promotes, while an RNA eraser (FTO) inhibits, the function of HNRNPA2B1 (Wang L. et al., 2019). The m6A modification influences the function of retinoic acid-induced gene I (RIG-I). RIG-I plays a key role in recognizing viral infection, and its activated conformer engages the adaptor mitochondrial antiviral signaling protein (MAVS) to mediate the activation of transcription factors and interferon-stimulated gene (ISG) (Durbin et al., 2016). RNAs containing modified nucleotides interrupt signaling at early steps of the RIG-I-like innate immune activation pathway, and nucleotide modifications with similar chemical structures are organized into classes that suppress or evade innate immune signaling (Durbin et al., 2016). This result is consistent with the mechanism we discussed above in DCs, in which the innate immune system may selectively respond to invading pathogenic nucleic acids by detecting RNA lacking modification (Karikó et al., 2005). For example, human metapneumovirus (HMPV) RNA is modified by m6A, which promotes HMPV replication and gene expression. However, if the m6A modification of HMPV RNA is demethylated, the production of IFN I is increased by the high expression of RIG-I (Lu M. et al., 2020). Thus, if viruses or other invading pathogens acquire m6A in their RNA to mimic cellular RNA, they might avoid detection by the innate immune system (Lu M. et al., 2020).

In addition, the m6A modification of ISGs is logical. The m6A machinery, such as METTL3, YTHDF2, and YTHDF3, has been reported to regulate IFN β levels *via* the m6A modification of ISGs (Winkler et al., 2019a, b; Zhang et al., 2019c). YTHDF2 assists ISG20 in degrading HBV transcripts by selectively recognizing and binding HBV transcripts with the m6A modification (Imam et al., 2020), as the m6A modification participates in IFN α-induced viral RNA degradation. A study conducted by Rubio R. M. et al. revealed that the writer METTL14 and eraser ALKBH5 negatively regulate IFN β production through the m6A modification of the coding sequence and the 3′ untranslated region of the IFN β mRNA (Rubio et al., 2018).

Methylation of host non-coding RNAs also exerts an effect. The m6A modification of lncRNAs plays an important role in antiviral innate immunity based on IFN I signaling (Wang et al., 2020). Studies have revealed that m6A on circular RNA (circRNA) inhibits innate immunity (Chen Y. G. et al., 2019). CircRNAs lacking the m6A modification directly activate the RNA pattern recognition receptor RIG-I in the presence of lysine-63-linked polyubiquitin chains to cause filamentation of the adaptor protein MAVS and activation of the downstream transcription factor IRF3, potentially inducing IFN I production (Chen Y. G. et al., 2019).

### Role of RNA Methylation in the Antiviral Immune Response

RNA methylation also plays an important role in the antiviral immune response, which is regulated by two systems: the innate immune response and adaptive immune response. In addition to IFN I production, the m6A modification has also been shown to regulate the innate immune response through other mechanisms. Toll-like receptors (TLRs) recognize viral RNA, which is an important information transmission process in the innate immune response (Karikó et al., 2005). Methylated RNA destroys the stability of double-stranded RNA bodies (Yang et al., 2020), which restrains the activation of TLRs (Kierzek and Kierzek, 2003).

RNA methylation is also important in specific antiviral immune responses. For example, in HIV-infected T cells, both host and viral RNA methylation levels are elevated (Lichinchi et al., 2016). Knockdown of METTL3 or METTL14 alone results in reduced viral replication, while ALKBH5 silencing results in significantly increased viral replication, indicating that m6A methylation exerts a positive regulatory effect on HIV replication (Lichinchi et al., 2016). YTHDF3 overexpressed in CD4^+^ T cells recognizes the m6A modification on HIV and limits its infection (Jurczyszak et al., 2020). YTHDF1-3 also restrains HIV-1 reverse transcriptase (Tirumuru et al., 2016). In HIV-1-infected cells, 56 genes modified by m6A methylation were identified, 19 of which are related to HIV replication and mainly encode functional proteins. The m6A modification of these 56 genes is mainly involved in the T cell response to viral infection by changing their RNA metabolism (Lichinchi et al., 2016). At the same time, the m5C modification also plays a role in antiviral immunity. Experiments have proven that m5C regulates the splicing of HIV-1 mRNA and posttranscriptional function, which affects the expression of viral genes (Courtney et al., 2019). In a direct homologous RNAi screen of host factors regulating HIV-1 replication, the m5C methyltransferase NOP2/NSUN1 was found to limit HIV-1 replication in the nucleus. NOP2 is associated with the HIV-1 5′ LTR and interacts with the HIV-1 TAR RNA through its competition with HIV-1 Tat protein and contributes to the methylation of TAR m5C, which also proves that m5C methylation promotes HIV-1 transcription and virus entry into the incubation period (Kong et al., 2020). RNA chemical modification plays an important role not only in the specific immune response to HIV but also in the responses to other viruses, such as Kaposi’s sarcoma-associated herpes virus (KSHV), and m6A has become one of the new targets for the treatment of KSHV (Ye, 2017; Tan and Gao, 2018).

### RNA Methylation in Autoimmune Responses

SLE is a typical systemic autoimmune disease characterized by various autoantibodies, a loss of tolerance and tissue damage. While less evidence is available for the regulation of m6A in the autoimmune response, the role of m6A in autoimmunity is non-negligible. In patients with SLE, the levels of ALKBH5 mRNA are associated with anti-dsDNA and antinucleosome antibodies, which are typical autoantibodies detected in patients with SLE (Luo et al., 2020a). IL-17 participates in various autoimmune diseases and in autoantibody production in patients with SLE (Ma et al., 2020). Wang et al. (2017) found that NSun2 methylates the IL-17A mRNA at cytosine C466 *in vitro* and *in vivo*, which promotes the translation of IL-17. Evidence has shown that m6A RNA methylation participates in coeliac disease, a complex autoimmune disorder (Olazagoitia-Garmendia et al., 2021). Higher m6A methylation in the 5′ UTR of the XPO1 RNA results in increased XPO1 protein concentrations that lead to nuclear factor kappa B activation and inflammation (Wang et al., 2017). Although we did not obtain direct evidence for the relationship between RNA methylation and autoantibodies in patients with SLE, these findings suggested that the topic deserves in-depth study.

## RNA Methylation and Organ Damage in Patients With Sle

Evidence shows that the loss of tolerance and tissue damage are distinct processes. Tissue effects might be major contributors to organ damage in patients with SLE independent of the effects of blood cells (Tsokos et al., 2016). These effects might also be mediated by RNA methylation.

### Nephritis

Renal tubulointerstitial fibrosis is one of the typical features of chronic kidney disease (CKD). In individuals with lupus nephritis, interstitial fibrosis is associated with CKD progression (Morales et al., 2021). A mouse fibrotic kidney disease model induced by UUO exhibited significant increases in the total m6A level, and ALKBH5, the eraser, suppressed fibrosis in this model (Ning et al., 2020). In an *in vitro* study, overexpression of METTL3 and METTL14 increased m6A levels and subsequently increased p53 mRNA and protein levels in cisplatin-treated HK2 cells (Zhou et al., 2019).

### Skin

Damage to stem cells in follicles might be one process leading to permanent loss of follicles in patients with SLE or cutaneous lupus erythematosus (CLE) (Al-Refu and Goodfield, 2009), which might also be regulated by RNA methylation. RNA methyltransferase (NSUN2) is required to balance stem cell self-renewal and differentiation in skin (Blanco et al., 2011). Compared with wild-type mice, an increase in the number of quiescent bulge stem cells was observed in *Misu*^–/^*^–^* mice, along with delayed exit from the bulge, increased self-renewal, and aberrant hair cycling (Blanco et al., 2011).

### Bone

Mesenchymal stem cells originally isolated from the bone marrow stoma are multipotent and possess strong immunomodulatory activities that interact with multiple immune cells, including DCs, neutrophils, NK cells, T cells, and B cells. Bone marrow-derived mesenchymal stem cells (BM-MSCs) from patients with SLE exhibit defective immune regulation, which might contribute to the imbalance between Treg and Th17 cells in patients with SLE (Geng et al., 2020). On the other hand, the capacity of osteogenic differentiation of BM-MSCs from patients with SLE is reduced compared with cells from healthy controls, which contributes to osteoporosis in patients with SLE (Tang et al., 2013). This outcome might be due to increased IFN β production and activation of the NF-κB pathway in BM-MSCs from patients with SLE (Tang et al., 2013; Gao et al., 2020). According to recent studies, METTL3 silencing reduces mA methylation levels and inhibits the osteogenic differentiation of bone marrow-derived mesenchymal stem cells (Wu et al., 2018; Yan et al., 2020). Thus, the METTL3-mediated m6A modification might induce the dysfunction of BM-MSCs in patients with SLE.

## Conclusion

We discussed the role of RNA methylation in the pathogenesis of SLE, including innate immunity and adaptive immunity. Although the evidence has generally indicated a potential relationship between RNA methylation and SLE, few studies have interpreted a direct relationship between them. Therefore, in the next step, we must investigate the direct relationship by quantitatively measuring m6A levels and their association with these existing mechanisms to establish a stronger causal link. Moreover, we investigated the function of RNA methylation in a cohort with SLE to obtain more biomarkers for the diagnosis, treatment, and prognosis of SLE and related complications. Further in-depth research on RNA methylation may clarify the pathogenesis of SLE and provide additional insights into diagnostic and therapeutic strategies.

## Author Contributions

XLV and XLI analyzed the literature and studies and wrote the manuscript. MZ, HW, and WZ assisted with constructing figures and polishing the language. QL and XC revised the manuscript. All authors listed have made substantial, direct and intellectual contributions to the work, and approved the article for publication.

## Conflict of Interest

The authors declare that the research was conducted in the absence of any commercial or financial relationships that could be construed as a potential conflict of interest.
